# Oral Health Status and Treatment Needs among Hemophilic Children in Hyderabad, Telangana, India

**DOI:** 10.5005/jp-journals-10005-1585

**Published:** 2019

**Authors:** Kola Srikanth Reddy, N Venugopal Reddy, P Niharika, M Ajay Reddy, V Danaeswari, MD Noorjahan

**Affiliations:** 1–6 Department of Pedodontics and Preventive Dentistry, Army College of Dental Science, Hyderabad, Telangana, India

**Keywords:** Children, Hemophilia, Oral health, Treatment needs

## Abstract

**Aims and objectives:**

The aim of this study is to evaluate and compare the dental caries stratus and treatment needs of hemophilic children with that of healthy and normal children.

**Materials and methods:**

A descriptive cross-sectional study was conducted on 60 subjects with age ranging from 7–16 years attending and registering their names, residential address, parental occupation, and other criteria in Hemophilic Society at the Telangana state. The oral hygiene status was recorded by using oral hygiene index-simplified (OHI-S). Teeth affected by dental caries and teeth restored/extracted as sequale of dental caries were assessed using decayed, missed, filled tooth (DEFT) and DMFT for primary and permanent dentition, respectively.

**Statistical analysis:**

Data were analyzed by means of SPSS, version 21.0, and the Chi-square test and the Kruskal–Wallis test.

**Results:**

The mean oral hygiene index simplified scores were 1.28 in 7–9 years and 1.87 in 13–16 years, respectively. The mean DMFT + DEFT of 7–9 years was 4.76 and for 13–16 years was 3.11 highest mean DMFT + DEFT 4.76 was recorded in the 7–9 years age group.

**Conclusion:**

The present study showed that oral hygiene status of hemophilic children was poor and treatment requirement was high among hemophilic children. The overall prevalence of dental caries was 73.3% and the treatment needs were 93.90%.

**How to cite this article:**

Reddy KS, Reddy NV, *et al*. Oral Health Status and Treatment Needs among Hemophilic Children in Hyderabad, Telangana, India. Int J Clin Pediatr Dent 2019;12(1):30–32.

## INTRODUCTION

Hemophilia is a hereditary disease of the mechanism of bleeding clinically manifested as a tendency to bleed which presents serious challenges in a dental practice.^1^

As inherited bleeding disorders account for 1 in 10,000 live birth and hemophilia represents the majority of such disorders, they are the priority group for dental care.^2^

Hemophilic children must be thought of as special patients. The bleeding tendency and fear of bleed may have a negative effect on preventive dental care of patients with hemophilia both at home and at dental appointments. Although there have been a number of studies regarding oral, surgical, and periodontal management of hemophilic patients, there are few studies regarding the oral health status in such patients.

Hemophilia A is the deficiency of factor VIII, it is most common, and hemophilia B also called the Christmas disease is the deficiency of factor IX, hemophilia B is affecting approximately 1 in 7,500 males and Von Willbrand disease. Hemophilia C, involving factor XI, is very rare but much milder than hemophilia C, involving factor XI, is very rare but much milder than hemophilia A or hemophilia B. Other factor defecies, such as those of factor II, V, and XII (1 case per 1 million) and factor VII (1 case per 500,000). Are rare and extremely un common.

As the age increase, the child's physical activity naturally increases, which results in more exposure to trauma, especially in hemophiliacs.

Oral health instructions create awareness of the need to return regularly for examination, professional prophylaxis, and treatment. Appropriate dental care is necessary for these individuals; however, the issue gains more importance as dental care affects the general health of hemophilic patients.^3^

The aim of the present study is to assess the oral hygiene status, the prevalence of dental caries, and treatment needs in children referred to the Hyderabad Hemophilic Society, Telangana state.

## MATERIALS AND METHODOLOGY

### Study Design

A descriptive cross-sectional study was conducted at the Department of Pedodontics and Preventive Dentistry, Mamata Dental College, Khammam, Telangana, India.

The sampling procedure involved multistage stratified sampling,^4^ where the Hyderabad is divided into five strata, namely Hyderabad central, Hyd southern, northern, eastern, and western.

Eligible children were selected randomly from a list obtained from Hyderabad Hemophilic Society records. Age eligibility requires that the children fall into the appropriate age at the time of sampling.

### Study Sample

A total of 60 children, age ranging from 7 to 16 years, were selected for this study.

### Research Methodology

Data collection instrument included the number of data types and different data collection instruments. It consisted of retrospective data collection and concurrent data.

### Study Questionnaire

A questionnaire included questions about the presence of other hemophilic members of family, type of dietary habits, frequency of tooth brushing, education and economic level of parents, and parent's oral hygiene habits. The draft of the questionnaire was reviewed by the panel of experts which included faculty members from the Pedodontics and Preventive Dentistry, Public Health Dentistry.

### Inclusion and Exclusion Criteria

The children suffering from hemophilia were only included in the study. Other hematological disorders and systemic diseases were excluded.

### Measurements of Oral Hygiene Status and Dental Caries

Oral hygiene status was recorded by using OHI-S^5^ as reported by Grene and Vermillon.

Teeth affected by dental caries. The teeth restored/extracted as sequale of dental caries were assessed using decayed, missed, filled tooth (DEFT), DMFT^6^ for primary and permanent dentition respectively was recorded by using WHO criteria in 1997. Missing primary incisor teeth for children aged 7 years and older were considered exfoliated and were not included in the “m” component, as was missing primary canines or molars in children aged 9 or 10 years. When permanent teeth were missing, a history of the reason for tooth extraction was obtained to ensure that orthodontic extractions were not included in the “M” component.

### Statistical Analysis

Statistical analysis was performed using the Statistical Package of Social Science (version 21; SPSS Inc., Chicago, IL, USA). Frequency tables were computed. The results are presented in mean ± standard deviation and percentages. The observations were statistically analyzed using the Chi-square test and the Kruskal–Wallis test. *p* < 0.05 was considered statically significant.

## RESULTS

Table 1 shows that a total of 60 children were examined, children belong to the age group of 7–9 years were 21, 10–12 years were 20, and 13–16 years 19.

Table 2 shows that a total of 93.90% of children require dental treatments. Children within the age of 7–9 years, 80.95% require preventive care, 33.3% fissure sealant, 23.80% extractions, 47.6% crowns and the age group of 10–12 years requires 100% restorations, 20% extractions, 65% pulp care, 7–9 years age group children require more dental interventions than compared to the 10–12 years and 13–16 years age group children.

Table 3 shows that the difference between the two groups like DMFT and DEFT; the *p* value is 0.024; *p* > 0.05, it is not statistically significant; the OHI-S between two groups, the *p* value is 0.043; *p* > 0.05, it is not statistically significant.

Table 4 shows that the mean DMFT/DEFT of 7–9 years was 4.76 and for 10–12 years was 3.75 and for 13–16 years was 3.11. The highest DMFT/DEFT scores were recorded in 7–9 years age group's children. The mean oral hygiene index simplified score was 1.28 in 7–9 years and 1.44 in 10–12 years and 1.87 in 13–16 years, respectively. The poor oral health status was recorded in the 13–16 years age group.

**Table 1:** Sample distribution according to the age group

*Age group*	*n*
7–9 years	21
10–12 years	20
13–16 years	19
Total (*N*)	60

**Table 2:** Treatments needs of study population according to the age group

*Treatment*	*7–9 years*	*10–12 years*	*13–16 years*
Preventive care	17 (80.95%)	12 (60%)	8 (42.10%)
Fissure sealant	7 (33.3%)	5 (25%)	3 (15.78%)
Restoration	18 (85.7%)	20 (100%)	16 (83.1%)
Crown	10 (47.6%)	7 (35%)	8 (42.20%)
Pulp care	12 (57.14%)	13 (65%)	12 (63.15%)
Extraction	5 (23.80%)	4 (20%)	1 (5.26%)

**Table 3:** Intra-analysis of mean DMFT and mean DEFT study population

	*Sum of squares*	*df*	*Mean square*	*F*	*p value*
DMFT	42.241	2	21.121	11.610	0.000
Between groups	103.69	57	1.819		
Total	145.93	59			
DEFT	138.44	2	28.087	28.087	0.000
Between groups	140.48	57			
Total	278.93	59			
DMFT + DEFT	28.051	14.026	3.970	3.970	0.024
Between groups	201.34	3.532			
Total	229–240				
OHI-S	3.705	1.85	3.314	3.314	0.043
Between groups	31.857	0.559			
Total	35.562				

* *p* > 0.005 is statically not significant

**Table 4:** DMFT and DEFT group

	*n*	*Mean*	*Standard deviation*	*Standard error*
DMFT + DEFT				
7–9 years	21	4.76	2.343	0.511
10–12 years	20	3.75	1.743	0.390
13–16 years	19	3.11	1.370	0.314
Total	60	3.90	1.972	0.255
OHI-S				
7–9 years	21	1.28	0.763	0.167
10–12 years	20	1.44	0.586	0.131
13–16 years	19	1.87	0.872	0.200
Total	60	1.52	0.776	0.100

* *p* > 0.005 is statistically not significant

Table 5 shows that Out of 60 subjects 44 children were affected with dental caries. Overall prevalence of dental caries is 73.3% and patients showed poor oral hygiene status was 73.7%.

## DISCUSSION

The present study recorded the overall caries’ prevalence as 73.3% with a mean DEFT and DMFT of 4.76 in the 7–9 years age group and 3.75 in the 10–12 years age group, and 3.11 in the 13–16 years age group. According to the WHO, the mean DMFT at the age of 12 years should not be more than 3.^7^

**Table 5:** Prevalence of dental caries according to the age group

*Age group count % within age group*	*DMFT and DEFT group*	
	*Normal (%)*	*Abnormal (%)*
7–9 years	3 (14.3)	18 (85.7)
10–12 years	6 (30.6)	14 (70.0)
12–16 years	7 (36.8)	12 (63.2)
Total	16 (26.7)	44 (73.3)

In a study, Boyd and Kinirons^8^ observed a slightly higher prevalence of dental caries in the primary dentition of hemophilic children compare to control. Similarly, in a study by Kabil et al.,^9^ DMFT and DEFT of hemophilic children were significantly higher than those of the non-hemophilic in Egypt.

Our results are in contrast with the study conducted by Sonbol et al.^10^ where significantly greater proportion of children with severe hemophilia were caries free compared with controls. Both the DMFS and DMFT were significantly greater in the controls compared with the hemophilic group. This may be due to the fact that there patients received dental care as dental department was situated next to the out patient hematology consulting room and the dental aspect of the service was considered as an integral part of the hematology visit.

In the present study, the oral hygiene index simplified was scored as poor in 13–16 years (1.87), 10–12 years (1.44), and 7–9 years (1.28) age group. These findings were similar to a study conducted in Poland where the oral hygiene index of hemophilic children was recorded as worse when compared to controls.^11^

Our results are in contrast with a study conducted by Zaliuniene et al.^12^ showing that the overall findings were better in deciduous dentitions, i.e., less overall caries experience and lower dental treatment needs were observed in children with hemophilia as compared to their healthy counterparts. However, there was a significant difference observed when permanent dentitions were compared between the hemophiliacs and controls.

In the present study, the mean DMFT of 4.76 in 7–9 years, 3.75 in 10–12 years, and 3.11 in the 13–16 years age group, which was not in agreement with the study conducted by Evangelista^13^ where the DMFT score was 0.9, which might be due to the early intervention of dental caries. The present study was not in accordance with a study by Rodrigues et al.^14^ who recorded a mean DEFT of 2.00.

In the present study, the crown requirement study was reported as the 7–9-year age group and 35% in the 10–12-year age group. The probable reason could be that as age increases, the prevalence of caries progression increases and the requirement of treatment needs increases, except for crowns, it decreased as age increases dental caries in mixed dentition, the spread of caries process is faster, so the requirement of crown is more in 7–9 years old children than in 10–12 years.

In the present study, the overall treatment required was 93.90%, in that 80.95% required preventive care, 33.33% fissure sealant, 100% surface fillings, 23.80% extraction, 63.15% pulp care, and 47.6% crown. The results of the present study were in accordance with the study reported by Sudhanshu et al.^7^

## CONCLUSION

Our data show that high dental caries was seen in 7–9 years old hemophilic children Hyderabad. The present study showed the oral hygiene status was poor and the dental caries, the prevalence was 73.3% and the 93.3% required treatment needs.

## LIMITATIONS

Further studies are recommended to better understand oral health of hemophilic children, and a longitudinal study with a large size is needed.

## RECOMMENDATIONS

Good oral health is essential to improve individual overall health and wellbeing. This overview points toward developing immediate and effective oral health promotional and interventional strategies to combat the diseases in this special group.
